# Trends of Ankylosing Spondylitis Ossification

**DOI:** 10.4021/jocmr1409w

**Published:** 2013-06-21

**Authors:** Alexander P Rozin

**Affiliations:** a B. Shine Department of Rheumatology. Rambam Health Care Campus and Technion. Haifa, Israel

## To the Editor

**Figure 1:**
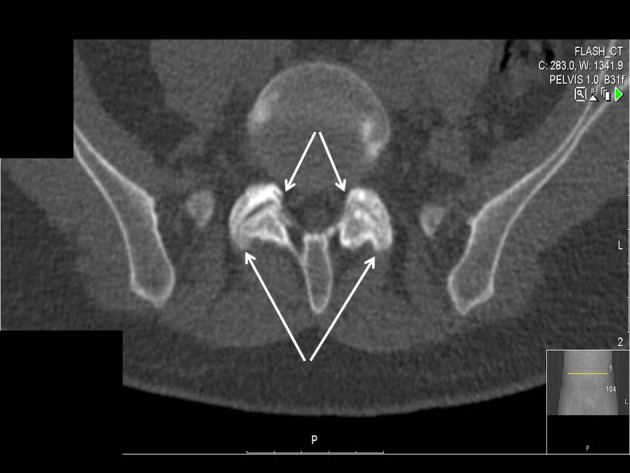
Low DKK-1/Wnts ration results in osteoblast induced marginal ossification of anulus fibrosus, ligaments and capsules (arrows) in patients with spondyloarthropathy.

**Figure 2:**
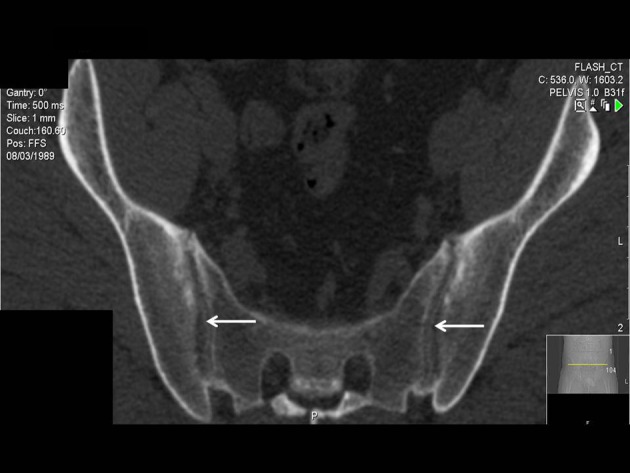
We met central SIJ ossification with free joint margins, which looks as yet an unreported pathway of the SpA ossification in 24-year-old man with 1.5 year history of inflammatory back and buttock pain and normal serum Ca and PTH.

Different inflammatory joint diseases have distinct patterns of bone damage with pronounced erosions without repair in rheumatoid arthritis (RA), a combination of bone destruction and bone formation in psoriatic arthritis (PsA), and dominant new bone formation in non-psoriatic spondyloarthritis (SpA). Wnts proteins are important for the development and maintenance of bone by inducing the differentiation and maturation of precursor osteoblasts to active osteoblasts. Dickkopf-1 (DKK-1) protein antagonizes Wnts by binding to the LRP receptor on the target cells, resulting in internalization of the receptor and inhibition of Wnt-mediated canonical signaling [1]. Consistently, serum DKK-1 levels are elevated in human RA but low in axial SpA. Low DKK-1/Wnts result in osteoblast induced marginal ossification of anulus fibrosus, ligaments and capsules in SpA patients (Fig. 1) and high DKK-1/Wnts induce RANKL-osteoclast mediated erosive bone damage in RA patients. We met central SIJ chondrocalcinosis like ossification with free joint margins, which looks as yet an unreported pathway of the ossification in SpA in 24 year old man with 1.5 year history of inflammatory back and buttock pain and normal serum Ca and PTH. Endochondral ossification is one of the two essential processes during fetal development along with intramembranous ossification. The predominant forms of calcifications: calcium pyrophosphate dihydrate (CPPD) and basic calcium phosphate (BCP) have been recently studied but not in SIJ fibrocartilage [2]. Recently the positive correlation between cartilage calcification and aging has been accurately established [3]. Age related changes in cartilage water and proteoglycan content may play a role as well as lower level of TGF-beta with stimulation of production inorganic phosphate similarly inflammatory related [4, 5]. We propose vascular impairments associated with cartilage hypoxia in inflammatory joint. That is followed by deep layer cartilage necrosis, releasing basic phospate interacting with Ca++ forming crystal deposits (Fig. 2).
